# Gait Rehabilitation Using Functional Electrical Stimulation Induces Changes in Ankle Muscle Coordination in Stroke Survivors: A Preliminary Study

**DOI:** 10.3389/fneur.2018.01127

**Published:** 2018-12-20

**Authors:** Jessica L. Allen, Lena H. Ting, Trisha M. Kesar

**Affiliations:** ^1^Department of Chemical and Biomedical Engineering, West Virginia University, Morgantown, WV, United States; ^2^Division of Physical Therapy, Department of Rehabilitation Medicine, Emory University School of Medicine, Atlanta, GA, United States; ^3^Wallace H. Coulter Department of Biomedical Engineering, Emory University and Georgia Institute of Technology, Atlanta, GA, United States

**Keywords:** walking, functional electrical stimulation (FES), electromyography (EMG), neuromechanics, biomechanics, gait rehabilitation

## Abstract

**Background:** Previous studies have demonstrated that post-stroke gait rehabilitation combining functional electrical stimulation (FES) applied to the ankle muscles during fast treadmill walking (FastFES) improves gait biomechanics and clinical walking function. However, there is considerable inter-individual variability in response to FastFES. Although FastFES aims to sculpt ankle muscle coordination, whether changes in ankle muscle activity underlie observed gait improvements is unknown. The aim of this study was to investigate three cases illustrating how FastFES modulates ankle muscle recruitment during walking.

**Methods:** We conducted a preliminary case series study on three individuals (53–70 y; 2 M; 35–60 months post-stroke; 19–22 lower extremity Fugl-Meyer) who participated in 18 sessions of FastFES (3 sessions/week; ClinicalTrials.gov: NCT01668602). Clinical walking function (speed, 6-min walk test, and Timed-Up-and-Go test), gait biomechanics (paretic propulsion and ankle angle at initial-contact), and plantarflexor (soleus)/dorsiflexor (tibialis anterior) muscle recruitment were assessed pre- and post-FastFES while walking without stimulation.

**Results:**Two participants (R1, R2) were categorized as responders based on improvements in clinical walking function. Consistent with heterogeneity of clinical and biomechanical changes commonly observed following gait rehabilitation, how muscle activity was altered with FastFES differed between responders. R1 exhibited improved plantarflexor recruitment during stance accompanied by increased paretic propulsion. R2 exhibited improved dorsiflexor recruitment during swing accompanied by improved paretic ankle angle at initial-contact. In contrast, the third participant (NR1), classified as a non-responder, demonstrated increased ankle muscle activity during inappropriate phases of the gait cycle. Across all participants, there was a positive relationship between increased walking speeds after FastFES and reduced SOL/TA muscle coactivation.

**Conclusion:**Our preliminary case series study is the first to demonstrate that improvements in ankle plantarflexor and dorsiflexor muscle recruitment (muscles targeted by FastFES) accompanied improvements in gait biomechanics and walking function following FastFES in individuals post-stroke. Our results also suggest that inducing more appropriate (i.e., reduced) ankle plantar/dorsi-flexor muscle coactivation may be an important neuromuscular mechanism underlying improvements in gait function after FastFES training, suggesting that pre-treatment ankle muscle status could be used for inclusion into FastFES. The findings of this case-series study, albeit preliminary, provide the rationale and foundations for larger-sample studies using similar methodology.

## Introduction

Characterizing changes in muscle coordination after post-stroke gait rehabilitation may help identify neuromuscular mechanisms driving rehabilitation-induced improvements in walking function, and inform the effective prescription of gait rehabilitation to match individual-specific impairments. Currently, there is considerable inter-individual variability in clinical responses to gait rehabilitation, with often only 50% of participants achieving clinically-meaningful improvements (1). To improve rehabilitation prescription, we must understand the specific mechanisms underlying improvements in walking function for any given intervention. Clinical measures of walking function such as Timed Up and Go test or timed gait speed tests lack the sensitivity to differentiate true restitution of gait impairments (e.g., improved propulsive force generation from paretic ankle plantarflexor muscles) from maladaptive, energy inefficient compensations (e.g., increased contribution of non-paretic leg to forward propulsion, thus worsening inter-limb propulsive asymmetry). Biomechanical measures are advantageous for evaluating gait impairments and gait quality, but alone cannot differentiate between different muscle recruitment patterns that may underlie the same movement pattern (2, 3). Systematic examination of changes in muscle coordination, in conjunction with clinical function and biomechanics, can markedly enhance our understanding of rehabilitation mechanisms, enabling more targeted, and mechanistic prescription of post-stroke gait treatments.

Abnormal ankle muscle activity underlies many post-stroke gait impairments such as reduced paretic propulsion during stance and toe clearance during swing. Improving push-off from the paretic leg is a common target of rehabilitation interventions post-stroke, as deficits in paretic leg propulsion are related to poor walking performance and slow gait speed (4, 5). Appropriate ankle plantarflexor muscle recruitment, which is critical for generating propulsion (6, 7), is often impaired post-stroke (8–10). Similarly, foot-drop, a common post-stroke gait impairment limiting walking function (11, 12), is caused by weakness and inappropriate recruitment of ankle dorsiflexor muscles (13). Evaluation of activation deficits (using electromyography, EMG) in muscles targeted by an intervention can help determine whether abnormal muscle activation patterns underlying gait deficits are restored following rehabilitation.

FastFES, the combination of fast treadmill training and functional electrical stimulation (FES) of ankle muscles, is a novel post-stroke gait intervention that has been shown to improve walking speed, endurance, and energy efficiency post-stroke (14–16). FastFES targets paretic propulsion by using electrical stimulation to augment force generation of ankle plantarflexor muscles during terminal stance of walking (17). In addition, FastFES includes stimulation of paretic ankle dorsiflexor muscles during swing to correct foot-drop. Following 12-weeks of the FastFES intervention, increases in paretic propulsion were observed when walking *without* stimulation, and maintained 3 months post-training (14). This improved ability to generate propulsion from the paretic limb following FastFES was accompanied by increased walking speeds and function (14, 15, 18). Nevertheless, as is common with a majority of gait interventions, there was considerable inter-individual variability in the magnitude of FastFES-induced gait improvements, such that not all participants who underwent the intervention improved walking function (18). We posit that identifying neuromechanical mechanisms underlying improved walking function after FastFES can help identify candidates who are most likely to benefit, reducing variability in response to FastFES.

Because the FastFES gait intervention specifically aims to improve paretic ankle muscle recruitment, presumably changes in paretic ankle muscle recruitment drive the training-induced gait improvements. However, FastFES-induced changes in ankle muscle activation have not been assessed previously. It is currently unknown whether ankle muscle activity is actually changed after FastFES. Indirectly, predictions from gait simulations based solely on measured biomechanics suggest that improved plantarflexor recruitment underlies the increases in propulsion and walking speed observed after FastFES (19). But, direct evidence for improved plantarflexor muscle activity after FastFES through EMG recordings has not yet been demonstrated. Further, whether dorsiflexor recruitment is also altered by FastFES is also unknown. Long-term use of dorsiflexor stimulation alone to prevent foot-drop post-stroke has previously been shown to improve voluntary recruitment of the dorsiflexor muscles (20), but whether similar improvements are seen after FastFES, which involves delivery of FES to both dorsiflexors and the antagonist plantarflexor muscles, is unknown.

This study represents a first step in evaluating whether post-stroke gait rehabilitation combining FES applied to the ankle muscles while walking at fast speeds (FastFES) is effective at improving ankle muscle recruitment during walking. Whether intended changes in ankle muscle activity accompany improvements in walking function and gait biomechanics after FastFES has not been previously studied. We present here results from a preliminary case series utilizing single-subject research design on three individuals who participated in 18 sessions of the FastFES intervention.

## Materials and Methods

### Participants

Three individuals post-stroke participated in 18 sessions of FastFES training (3 sessions per week) (Table 1). Participants provided written informed consent before participating in accordance with the Declaration of Helsinki. The protocol was approved by institutional review boards at Emory University and Georgia Institute of Technology. This study was registered on ClinicalTrials.gov (NCT01668602).

**Table 1:** Participant demographics.

**Participant**	**Side of**	**Time since**	**Fugl-Meyer**
	**hemiparesis**	**stroke, mo**	**(LE) score**
R1	L	47	20
R2	L	35	19
NR1	R	60	22

### FastFES Gait Training

Before each training session, surface stimulation electrodes were attached to ankle dorsi- and plantarflexor muscles, and FES intensity was determined using procedures described in previous studies (15). Two footswitches were attached under the sole of the shoe of the paretic leg to determine gait events for closed-loop control of FES. During training, the fast training speed was selected after completion of a warm-up treadmill walking trial, as the fastest speed that the participant could maintain for 6-min without a break.

Each FastFES training session consisted of five 6-min bouts of treadmill walking at the fast speed (15). Each bout comprised of 1-min walking with FES followed by 1-min walking without FES repeated three times for a total of 6 min. FES was delivered to the ankle dorsiflexor muscles during the paretic swing phase. The subjects were informed that the purpose of the dorsiflexor FES was to assist with lifting their toes up while the foot is in the air. Plantarflexor FES was delivered during paretic terminal double support phase. The subjects were told that the purpose of plantarflexor FES was to assist with generating more push-off with their affected leg. The participants were instructed to use their own muscle activation to assist the FES, and to practice the walking patterns being trained by FES during intervening periods without FES. The intermittent FES delivery was designed to encourage motor learning of correct muscle recruitment patterns and discourage dependence on the FES as a neuroprosthetic. The participants were provided a 5-min seated break between bouts.

### Data Collection and Analysis

Each participant completed three separate evaluation sessions pre- and post-intervention to assess (1) clinical walking function, (2) gait biomechanics during treadmill walking, and (3) muscle activity during overground walking. Each evaluation session occurred within 2 weeks immediately preceding and following FastFES gait training. Changes from pre- to post-training were investigated within each individual using a pre/post single-subject research design (21).

#### Clinical Evaluation of Walking Function

Clinical tests included the 10-meter walk test for overground self-selected (SSWS) and fast walking speeds (FWS), 6-min walk test (6MWT) for overground walking endurance, and the Timed Up and Go test (TUG) for mobility and balance. Response to FastFES was determined based on changes in clinical walking function from pre- to post-training; if at least two measures exhibited improvements that exceeded the minimal clinically-important difference (MCID) or minimal detectable change (MDC), that participant was classified as a responder.

#### Gait Biomechanics Evaluation

Participants walked for 30-s at their self-selected speed without FES on a split-belt treadmill instrumented with force platforms embedded within each belt (Bertec Corp., Columbus, OH). To prevent the confounding influence of changes in speed on gait biomechanics, the post-training evaluation was conducted while participants walked at the pre-training self-selected speed. Twenty-four gait cycles per subject were analyzed at each testing session. Ground reaction forces (GRFs) were recorded at 1,000 Hz. Marker coordinate data were collected at 100 Hz using a seven-camera motion capture system (Vicon, Centennial, CO) from markers attached bilaterally to the thigh, shank, foot segments, and pelvis (17). Marker trajectories and GRF data were low-pass filtered at 6 Hz (4th order Butterworth) and 30 Hz, respectively. Vertical GRFs were used to identify gait cycles using a 20 N threshold. Lower limb kinematics were calculated in Visual3D (C-Motion, Germantown, MD). Data were time normalized to 100% of the paretic leg gait cycle. Primary biomechanical outcome measures were: (1) peak paretic propulsion (defined as peak anterior GRF during stance normalized to subject body weight) and (2) paretic ankle angle at heel-strike, which were calculated for each gait cycle. These two biomechanical outcomes were selected consistent with the impairments targeted by plantarflexor and dorsiflexor FES. Changes in biomechanical measures from pre- to post-training were examined using separate two-tailed *t*-tests (α = 0.05) for each participant. Effect sizes were also calculated with Cohen's d, calculated as differences in means between post-training and pre-training divided by stride-to-stride standard deviation at pre-training.

#### Muscle Activity Evaluation

**Figure 1:**
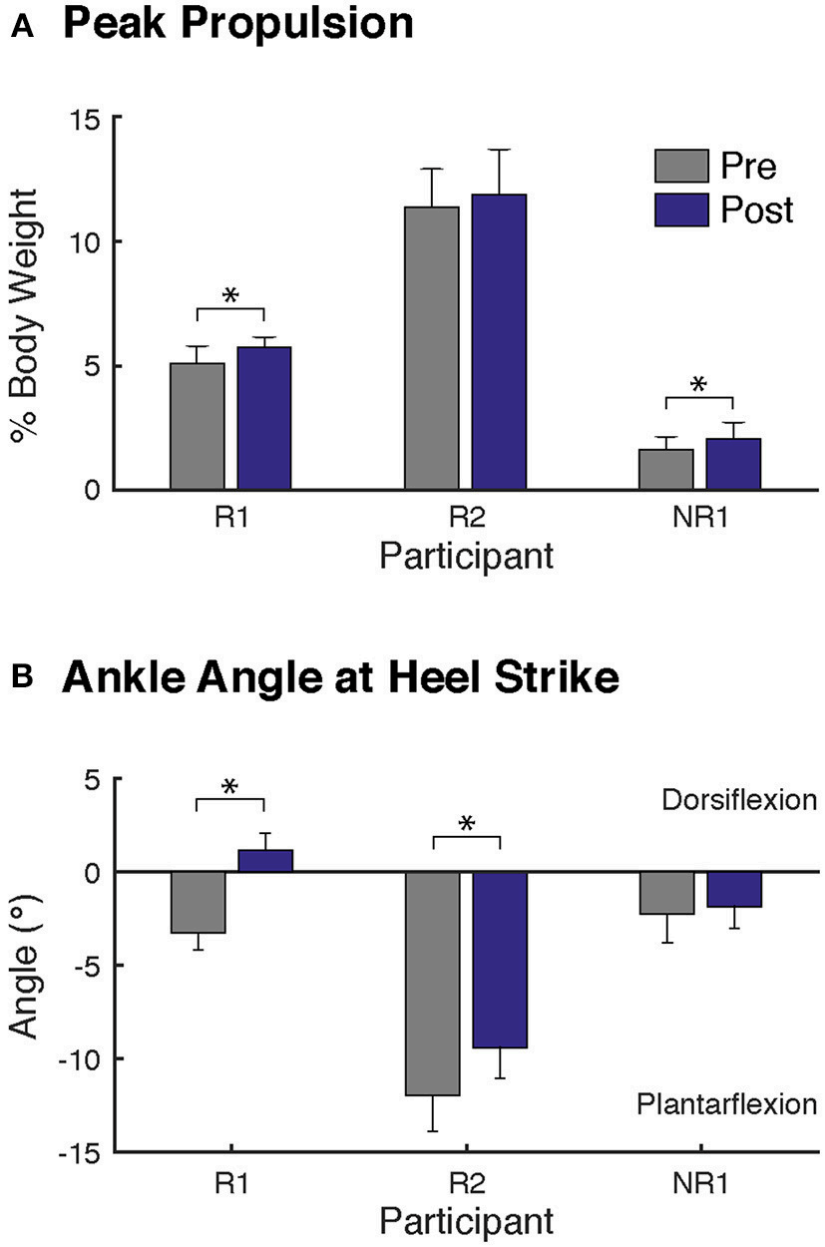
Changes in biomechanics at self-selected walking speed after FastFES. Two biomechanical outcomes were analyzed, consistent with the biomechanical parameters targeted by plantarflexor and dorsiflexor FES, **(A)** peak paretic propulsion, defined as peak anterior ground reaction force during stance, and **(B)** the paretic ankle angle at heel-strike, where positive values correspond to dorsiflexion. Participants R1 and NR1 demonstrated significant increase in paretic leg propulsion after FastFES **(A)**. Both responders (Participants R1 and R2) walking with greater dorsiflexion ankle angles at heel-strike after FastFES **(B)**. Note that to prevent the influence of changes in speed on gait biomechanics, the post-training evaluation was conducted while participants walked at their pre-training self-selected speed. (* denotes *p* ≤ 0.05).

Muscle activity during walking was assessed while participants walked overground for ~7.5 m at three different speeds (slow, SWS; self-selected, SSWS; and fast, FWS). At least three trials per speed were collected from each participant. Three-dimensional kinematics were measured using an 8-camera Vicon motion analysis system at 120 Hz and a custom 25-marker set that included head-arms-trunk, thigh, shank, and foot segments. Marker trajectories were low-pass filtered at 50 Hz. Surface electromyography (EMG) activity was recorded at 1,080 Hz from soleus (SOL) and tibialis anterior (TA) via a bipolar telemetered EMG system (Konigsburg Instruments, Pasadena, CA). After skin preparation by shaving (if necessary), local and gentle abrasion, and disinfection with alcohol, silver/silver chloride disc electrodes were attached to the skin with an inter-electrode distance of 2 cm by the same experimenter at each assessment. Photos of electrode placement taken at pre-training were used to aid in post-training electrode placement. EMG data were high-pass filtered at 35 Hz, de-meaned, rectified, and low-pass filtered at 10 Hz to obtain the linear envelope using custom MATLAB routines. EMG for each muscle was normalized to the maximum observed across all walking speeds. Walking speed for each trial was defined as the average velocity of the C7 marker in the middle 20 ft of the walkway. Gait events were identified based on heel-marker trajectories and EMG data were re-sampled at each 1% of the gait cycle. The first and last two steps of each trial were removed to avoid gait initiation and termination (Figure 1A).

Integrated EMG (iEMG) of each muscle during stance and swing (0–60 and 60–100% of the gait cycle, respectively) were calculated for each gait cycle within a trial.

Three different methods were used to evaluate changes in SOL and TA muscle activation following FastFES:

1. *Muscle activity during SSWS:* Changes in SOL and TA iEMG from pre- to post-training for each participant while walking at pre-training SSWS were examined using two-tailed *t*-tests (α = 0.05). Post-training trials were chosen in which walking speeds were within one standard deviation of the pre-training SSWS trials. Effect sizes were also calculated with Cohen's d, calculated as differences in means between post-training and pre-training divided by standard deviation at pre-training.
2. *Muscle co-activity across the gait cycle:* Coactivation of SOL and TA was quantified based on the percentage of overlapping area of activity between SOL and TA using the following formula (22):
  $$\begin{array}{l} \%\;Coactivation\;=\;100\;x\;\frac{2\;x\;overlapping\;area\;of\;activity\;}{area\;of\;TA\;activity\;+\;area\;of\;SOL\;activity} \end{array}$$
  Changes in SOL/TA coactivation from pre- to post-training for each speed condition (i.e., SWS, SSWS, FWS) were compared using two-sided *t*-tests (α = 0.05). Effect sizes were also calculated with Cohen's d, calculated as differences in means between post-training and pre-training divided by standard deviation at pre-training.
3. *Muscle activity modulation across walking speeds:* We examined the degree to which muscle activity varied across walking speeds using linear regression between iEMG and walking speed. The average iEMG was calculated for each trial across all walking speeds, resulting in a median of 9 iEMG values per muscle per subject at both pre- and post-training (Figure 3). Analysis was focused on SOL activity during stance and TA activity during swing. Separate regression equations were identified for each muscle in each participant, using the following model:
  $$\begin{array}{l} iEMG\;=\;\beta_{0}\;+\;\beta_{post}\times POST+\;speed\\ \times (\;\beta_{speed}+\;\beta_{post-speed}\;\times POST) \end{array}$$
  where:
  - *speed* is walking speed minus the average SSWS at pre-training
  - β_0_ is the intercept at pre-training
  - β_*post*_ is the change in intercept from pre- to post-training
  - β_*speed*_ is the slope at pre-training
  - β_*post*−*speed*_ is the change in slope from pre- to post-training
  - *POST* is an indicator variable set to 1 for post-training trials and 0 for pre-training trials.
    Each model was fit using the Matlab built-in function “regstats.” To test whether modulation (i.e., the slope) of muscle activity across walking speeds changed after rehabilitation, the following null hypothesis was evaluated with *t*-tests (α = 0.05):
    (1) $$\begin{array}{r} H_{0}:\beta_{post-speed}=0 \end{array}$$
    In this model, a positive β_*post*−*speed*_ corresponds to an increase in the modulation (or slope) of muscle activity across walking speeds after FastFES.

## Results

**Figure 2:**
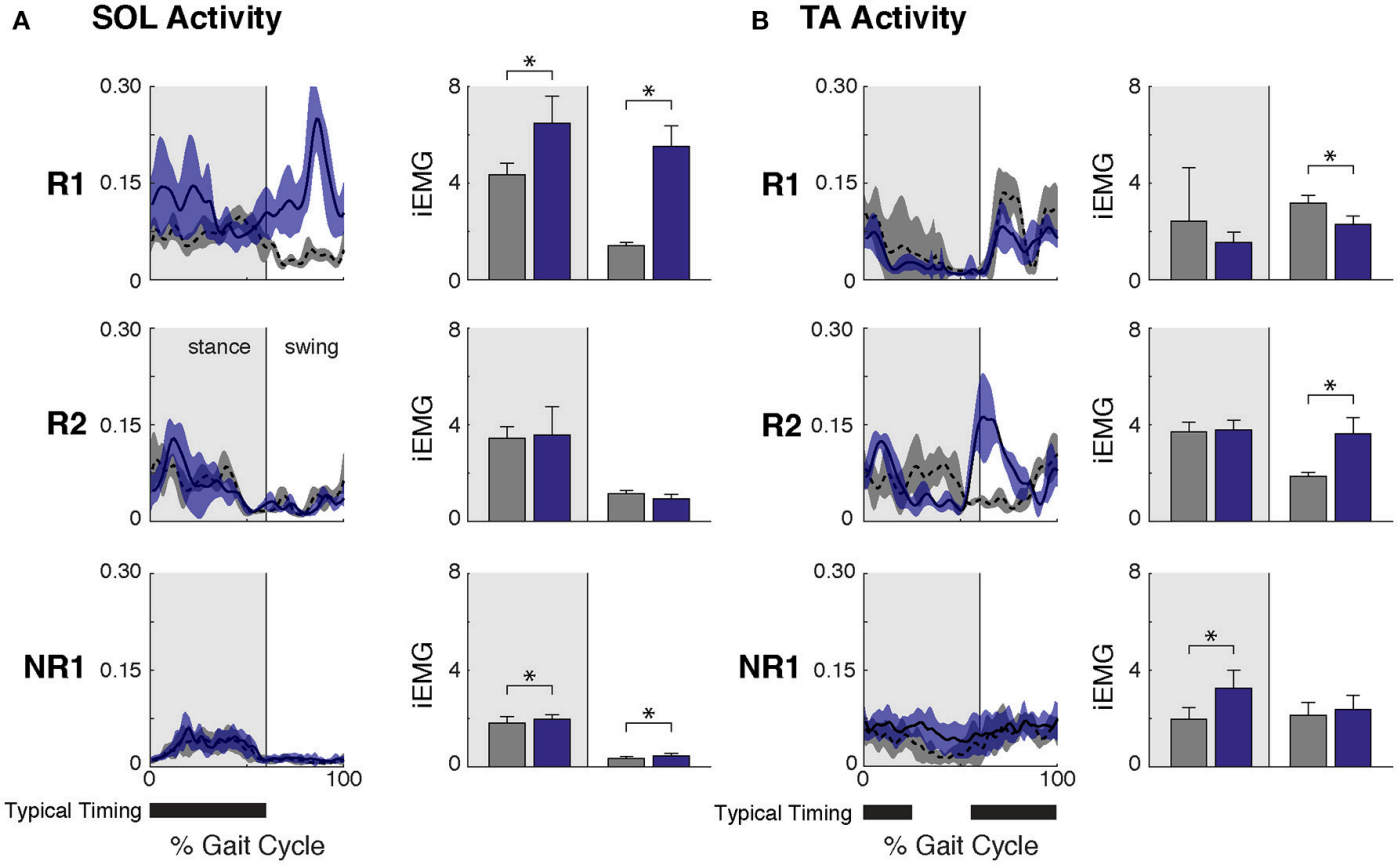
Ankle muscle activity while walking at self-selected speed before and after FastFES. **(A)** Soleus (SOL) muscle activity across the gait cycle (left, average ± one standard deviation) and integrated SOL activity during stance and swing phases (right, where stance phase was defined as 0–60% of the gait cycle and swing phase as 61–100% of the gait cycle). After FastFES Participant R1 demonstrated increased SOL recruitment during stance, and Participants R1 and NR1 had increased SOL activity during swing. **(B)** Tibialis anterior (TA) muscle activity across the gait cycle (left, average ± one standard deviation) and integrated TA activity during stance and swing phases (right). After FastFES, Participant R1 decreased TA activity during swing, Participants R2 increased TA activity during swing, and Participant NR1 had increased TA activity during stance. Note that to prevent the influence of changes in speed on muscle activity, post-training trials were selected with speeds matched to the pre-training self-selected walking speed. The average walking speeds across pre-training SSWS trials in participants R1, R2, and NR1 were 0.78 ± 0.07 m/s, 1.60 ± 0.20, and 0.47 ± 0.04, respectively, and for post-training at matched speeds were 0.75 ± 0.03 m/s, 1.70 ± 0.09, and 0.43 ± 0.02, respectively (* denotes *p* ≤ 0.05). Typical timing is from Perry (26).

**Figure 3:**
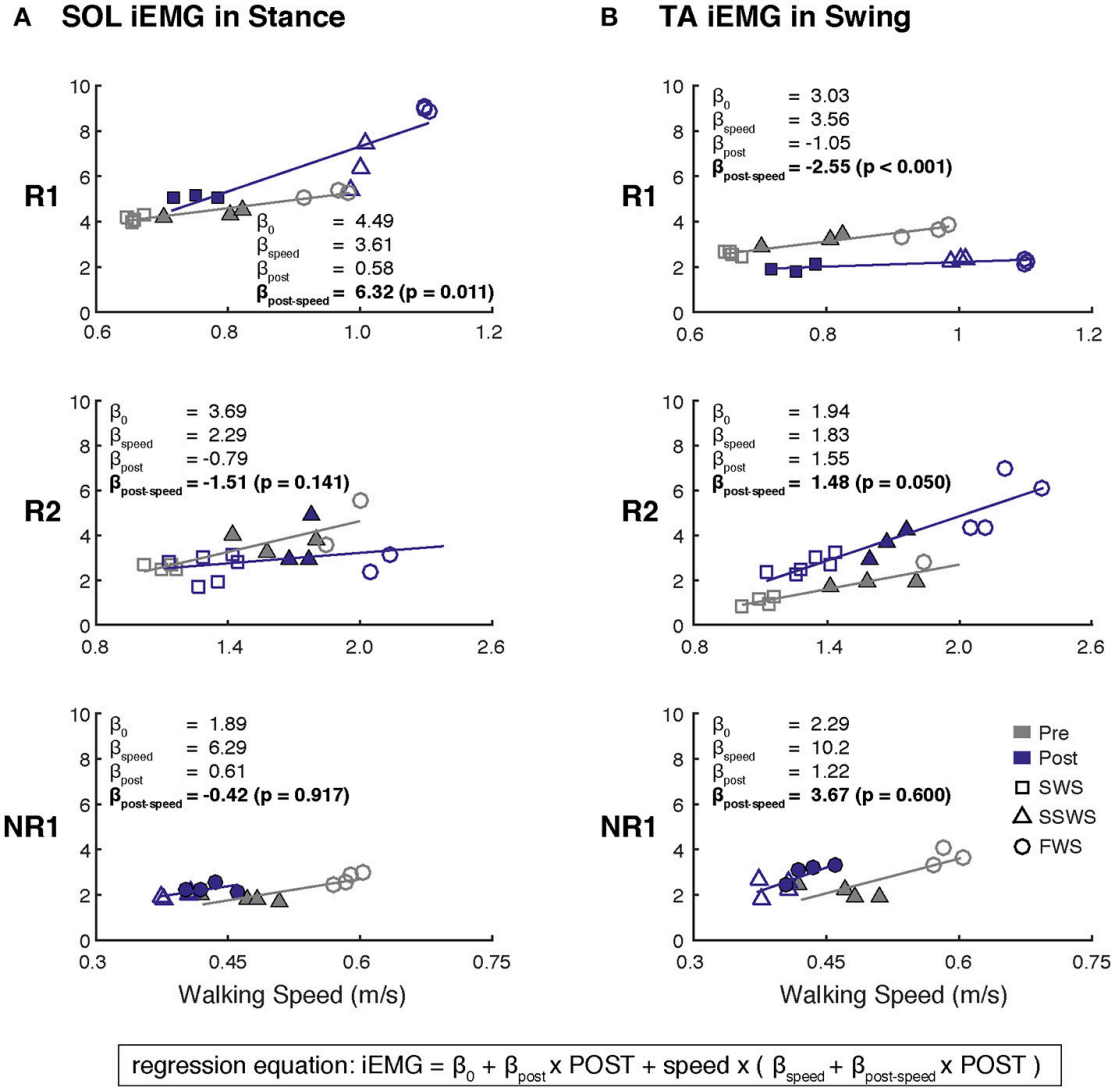
Changes in muscle activity modulation across walking speeds after FastFES for **(A)** Soleus (SOL) during stance and **(B)** Tibialis Anterier (TA) during swing. Linear regressions between iEMG and walking speed were generated before and after FastFES to examine the degree to which muscle activity was modulated with walking speed. A single regression equation was identified for each muscle in each participant, where the variable *speed* is walking speed minus the average self-selected walking speed at pre-training (light-colored triangles). Analysis was focused on whether the slopes of the regression for pre-training and post-training were significantly different (H_0_: β_post−speed_ = 0). Note that filled markers denote those trials with similar speeds pre- and post-training that were used in Figure 2. Participant R1 increased and decreased modulation of SOL and TA across walking, respectively. Participant R2 increased modulation of TA across walking speeds. Participant NR1 showed no changes in muscle activity modulation after FastFES (SWS, slow walking speed; SSWS, self-selected walking speed; FWS, fast walking speed).

**Figure 4:**
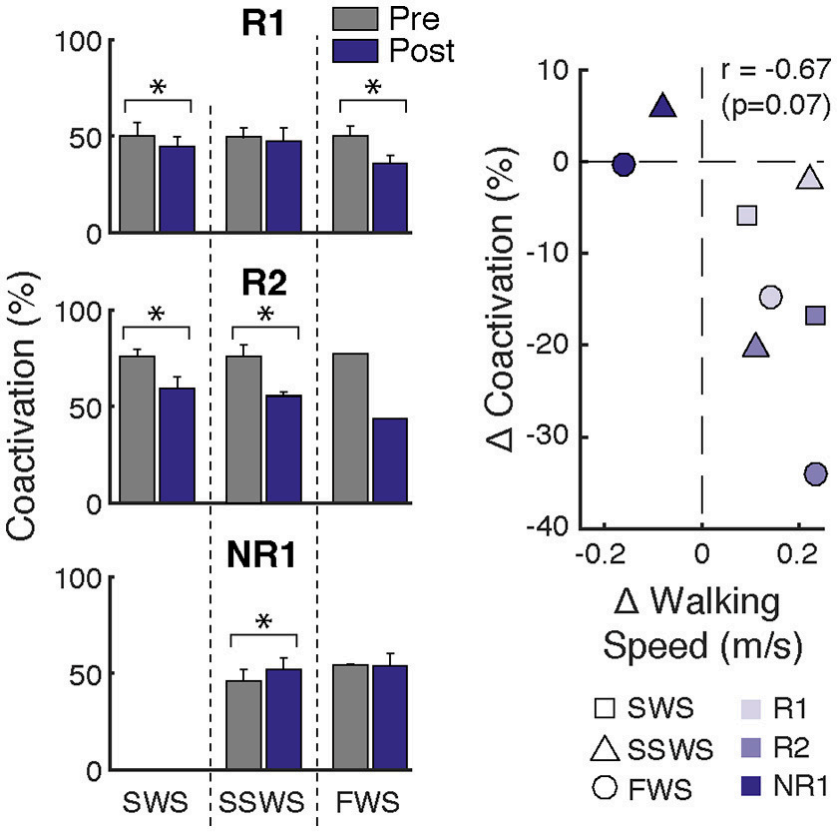
Changes in ankle muscle coactivation after FastFES. **(Left)** Ankle muscle coactivation between SOL and TA during the gait cycle while walking at slow-walking speeds (SWS), self-selected walking speeds (SSWS), and fast walking speeds (FWS) pre- and post-training. Coactivation was calculated as the percentage of overlapping muscle activity as in Winter 1990. Participants R1 and R2 demonstrated decreases in coactivation after FastFES, whereas Participant NR1 increased coactivation. **(Right)** Average change in coactivation vs. average change in walking speed at each speed condition. Correlation analysis revealed a trend for increased walking speeds post-training to be associated with reduced SOL/TA muscle coactivation (*r* = −0.67, *p* = 0.07). *p < 0.05.

Summary of changes from pre- to post-training in clinical walking function, gait biomechanics, and muscle activity are presented in Table 2, Figures 1–4, and detailed below for each participant.

**Table 2:** Clinical measures of gait before and after FastFES rehabilitation.

**Participant**		**SSWS (m/s)**	**FWS (m/s)**	**6MWT (m)**	**TUG (s)**
R1	Pre	0.70	0.91	310.8	15.88
	Post	0.98	1.14	379.2	13.78
	Change	0.28	0.24	68.4	−2.1
R2	Pre	1.56	1.87	520.7	6.55
	Post	1.18	2.05	580.3	5.55
	Change	−0.38	0.18	59.6	−1.00
NR1	Pre	0.42	0.50	164.9	24.88
	Post	0.32	0.32	139.4	31.10
	Change	−0.10	−0.18	−25.5	6.22

*SSWS, self-selected walking speed; FWS, fast walking speed; 6MWT, 6-min walk test; TUG, timed-up-and-go test. Where available, Minimal Clinically-Important Differences (MCIDs) for stroke are noted. SSWS/FWS, MCID = 0.16 m/s (23); 6MWT, MCID = 34.4 m (24). Minimal Detectable Change (MDC) for TUG in stroke = 2.9 s (25)*.

### Participant R1 (Responder)

Participant R1 exhibited improvements in three measures of clinical walking performance at post-training (Table 2): SSWS and FWS increased by 0.28 and 0.24 m/s, respectively [MCID = 0.16 m/s, (23)] and 6MWT distance increased by 68.4 m [MCID = 34.4 m, (24)]. Improvements in clinical walking function were accompanied by improved gait biomechanics (Figure 1); increased paretic propulsion (*p* = 0.002, ES = 0.94) and a shift from ankle plantarflexion to dorsiflexion at heel-strike (*p* < 0.001, ES = 4.81). These changes were accompanied by changes in both SOL and TA activity. When walking at pre-training SSWS, SOL iEMG was increased across the entire gait cycle (Figure 2; *p* < 0.001 and ES = 4.54 for stance, *p* < 0.001 and ES = 27.91 for swing) and TA iEMG was decreased during swing phase only (*p* = 0.204 for stance and ES = −0.40; *p* < 0.001 and ES = −2.69 for swing) from pre- to post-training. In addition, SOL/TA coactivation across the gait cycle (Figure 4) decreased from pre- to post-training during both SWS (*p* = 0.030, ES = −0.85) and FWS (*p* < 0.001, ES = −3.30) with no change during SSWS (*p* = 0.423, ES = −0.44). Across all walking speeds, Participant R1 showed increased modulation of SOL activation in stance and decreased modulation of TA activation during swing from pre- to post-training (Figure 3).

### Participant R2 (Responder)

Participant R2 exhibited improvements in two measures of clinical walking performance at post-training (Table 2): FWS was increased by 0.18 m/s [MCID = 0.16 m/s, (23)] and 6MWT distance increased by 59.6 m [MCID = 34.4 m, (24)]. R2 walked with decreased ankle plantarflexion at heel-strike after FastFES (*p* < 0.001, ES = 1.37) but exhibited no change in paretic propulsion (*p* = 0.423, ES = 0.31) (Figure 1). These changes were accompanied by changes in TA but not SOL muscle activity. When walking at pre-training SSWS, TA iEMG during swing was increased at post-training compared to pre-training (Figure 2; *p* < 0.001, ES = 12.15). No changes were identified for TA iEMG during stance (*p* = 0.760, ES = 0.23), SOL iEMG during stance (*p* = 0.789, ES = 0.30), or SOL iEMG during swing (*p* = 0.067, ES = −1.89). In addition, SOL/TA coactivation across the gait cycle was decreased (Figure 4; SWS: *p* = 0.007, ES = −4.67, SSWS: *p* = 0.003, ES = −3.56). Across all walking speeds, R2 exhibited increased modulation of TA iEMG but not SOL iEMG (Figure 3).

### Participant NR1 (Non-responder)

NR1 exhibited worse performance at post-training compared to pre-training in two measures of clinical walking performance (Table 2): FWS decreased by 0.18 m/s [MCID = 0.16 m/s, (23)] and TUG time increased by 6.22 s [MDC = 2.9 s, (25)]. Consistent with non-response, NR1 exhibited a small increase in peak paretic propulsion after FastFES (*p* = 0.050; ES = 0.81) but no change in ankle angle at heel-strike (*p* = 0.387; ES = 0.26) (Figure 1). These changes were accompanied by increases in SOL iEMG during stance (*p* = 0.049, ES = 0.62) and swing (*p* = 0.001, ES = 1.33) and TA iEMG during stance (*p* < 0.001, ES = 2.64) (Figure 2). No changes were identified for TA iEMG during swing (*p* = 0.264, ES = 0.44). However, SOL/TA coactivation (Figure 4) across the gait cycle slightly increased from pre- to post-training for SSWS (*p* = 0.014, ES = 0.95) and remained unchanged for FWS (*p* = 0.953, ES = −0.04). The modulation of SOL and TA across walking speeds did not change post-training (Figure 4).

## Discussion

Here, we examined changes in clinical walking function, gait biomechanics, and ankle muscle activity induced by the FastFES gait intervention, which utilizes targeted stimulation of the ankle plantarflexor and dorsiflexor muscles during appropriate phases of the gait cycle to retrain appropriate muscle recruitment and improve walking function. This is the first study to evaluate changes in the experimentally recorded activity from both ankle plantarflexor and dorsiflexor muscles after FastFES, in conjunction with measurement of training-induced changes in gait biomechanics and walking function. Consistent with heterogeneity of clinical effects previously observed following FastFES (18) and other gait treatments, only two of the three participants in this study were classified as responders. Improved clinical function in the responders was accompanied by changes in recruitment of both the ankle plantar- and dorsiflexors, but the responders showed nuanced differences in response-characteristics that were only captured through the use of biomechanics and muscle activation data. In general, the changes in ankle muscle activation observed after FastFES reflected observed improvements in biomechanics. However, the interactions among clinical walking function, gait biomechanics, and muscle activation are complex and multifaceted, as elucidated by our preliminary case series results.

### Responders Improved Clinical Walking Function After FastFES Through Different Mechanisms

#### Participant R1 Increased Propulsion Through Improved Ankle Plantarflexor Recruitment

Given that the plantarflexors are critical for forward propulsion generation and walking speed (6, 7), the ability to more strongly recruit SOL likely played a large role in Participant R1's increased walking speed post-training. Participant R1 walked faster (SSWS and FWS) and had increased endurance (6MWT) after completing the FastFES program. R1 is an example of the “classic” responder to FastFES whose faster walking speeds were accompanied by increased paretic leg propulsion (Figure 1A). As predicted by a prior musculoskeletal modeling simulation of FastFES (19), increased paretic leg propulsion observed in R1 was accompanied by improved SOL recruitment during stance (Figure 2A, top panel). R1 also exhibited increased modulation of SOL activity as a function of walking speed after FastFES (Figure 3A, top panel), suggesting that overall capacity to recruit SOL was improved.

However, R1 also demonstrated unanticipated changes in ankle muscle activity during swing phase: increased SOL activity and decreased TA activity. SOL is not typically active during swing phase (26) and the combination of increased SOL and decreased TA activity during swing is inconsistent with the small reduction of foot-drop observed post-training (increased dorsiflexion at heel-strike, Figure 1). A limitation to our current analysis is that gait biomechanics were assessed while walking on a treadmill whereas muscle activity was assessed in a different laboratory while walking overground, which may explain this inconsistency. Nevertheless, it is possible that the unintended changes in SOL and TA activity during swing phase may represent a compensatory or adaptive strategy during training in response to TA stimulation during swing phase. Identifying unintended changes in ankle activity may inform additional rehabilitation efforts to further improve walking function. For example, by capitalizing on baseline values of short-term training-induced changes in muscle activation and biomechanics, a subset of stroke survivors who demonstrate a positive response to plantarflexor FES but not dorsiflexor FES (such as R1) could potentially be prescribed plantarflexor stimulation only.

#### Participant R2 Improved Ankle Dorsiflexor Recruitment During Swing

Participant R2 demonstrates that that FastFES can also act to increase walking function through improved dorsiflexor recruitment for swing leg control. Similar to Participant R1, Participant R2 also walked faster (FWS, Table 2) and had increased endurance (6MWT) after FastFES training. However, Participant R1 and R2 presented very differently at pre-training in terms of both paretic propulsion and paretic ankle control (Figure 1). As such, R2 did not exhibit increased propulsion after FastFES or changes in SOL activity. Instead, R2 showed improvements in the swing phase, walking with a less plantarflexed ankle at heel-strike, i.e., reduction of foot-drop. This was accompanied by increased TA activity during swing phase at self-selected speed (Figure 2B, middle panel), increased modulation of TA activity across walking speeds (Figure 3B, middle panel), and decreased SOL/TA coactivation across the entire gait cycle (Figure 4).

Participant R2 also illustrates how muscle activity may be a more sensitive and specific tool for identifying motor control pathologies compared to clinical and biomechanical metrics, revealing beneficial effects of rehabilitation that may not be measured otherwise. R2 would be classified as a high-functioning stroke survivor at baseline based on clinical walking function (Table 2; e.g., walking at 1.4 m/s) and many gait rehabilitation studies may exclude this individual from participation. Despite high baseline clinical walking function, our EMG analysis revealed that R2 walked with improperly timed TA recruitment during gait prior to FastFES such that TA was coactivated with SOL during stance (Figure 2B). This abnormal coactivation was remediated after FastFES, such that the TA was more appropriately recruited during the swing phase. Muscle activation data demonstrated unique training-induced improvements in ankle motor control, which may have been ignored with the use of clinical or biomechanical outcomes alone. Thus, Participant R2's data showcase the important and complementary role that EMG coordination measures can play in clinical rehabilitation study design and decision-making.

### Factors Contributing to Non-response to FastFES Need More Investigation

Consistent with prior studies demonstrating heterogeneity in response to gait rehabilitation post-stroke, not all of our participants improved their walking function after FastFES. Although a small increase in propulsion was identified in Participant NR1 after FastFES (Figure 1), walking performance was not improved and NR1 walked *slower* at the post-training testing session (Table 2). This slower walking speed was accompanied by increased TA activity during stance and a small increase in SOL activity during swing, phases during which these muscles are not typically active and were not stimulated during training, such that SOL/TA coactivation over the gait cycle was increased after training. It is possible that the reduction in walking speed observed in NR1 after training was within the day to day fluctuations in pre-training walking speed; future studies should implement multiple testing sessions at both pre- and post-training to account for such day to day fluctuations. Nevertheless, NR1 would be considered a non-responder even if the slower walking speed observed post-training was within the variability of day to day fluctuations, because NR1 did not improve in any clinical measure examined in the current study.

One possibility to explain NR1's lack of response to FastFES training is that ankle muscle recruitment may not have been the primary cause of gait impairment in this participant and may be secondary to more proximal deficits. In such a case, a different type of FES strategy (e.g., stimulating proximal muscles such as quadriceps) may be more appropriately matched to NR1's underlying impairments. Alternatively, NR1's abnormal muscle activation patterns may be related to heightened spinal reflex excitability of ankle plantarflexor muscles, and amenable to restitution by interventions that specifically target hyper-reflexia and spasticity (27). Recording muscle activity from additional muscles and/or evaluating corticospinal excitability may aid in understanding NR1's baseline deficits. Participant NR1's data suggests the importance of identifying factors that may predict response to targeted gait rehabilitation interventions, to customize interventions to meet individual-specific gait deficits, and reduce the likelihood of non-response (28–30).

### FastFES May Reduce Ankle Muscle Coactivation

Although changes in ankle muscle activity after FastFES differed between participants, a pattern emerged for training-induced changes in ankle muscle coactivation (Figure 4). Both responders reduced SOL/TA coactivation after FastFES, indicating restitution of more normal physiological muscle activation coordination during gait. In contrast, the non-responder increased SOL/TA coactivation after training, indicating worsening of muscle activation coordination. To further investigate ankle muscle coactivation, the relationship between changes in SOL/TA coactivation and walking speed after FastFES was examined. *Post-hoc* Pearson's correlation analysis revealed a trend for increased walking speeds post-training being associated with reduced SOL/TA muscle coactivation (*r* = −0.67, *p* = 0.07; Figure 4, right panel). This suggest that inducing more appropriate (i.e., reduced) SOL/TA coactivation may be an important neuromuscular mechanism underlying improvements in gait function through FastFES training.

Stimulation of the ankle dorsi- and plantarflexors during FastFES may promote motor learning of more appropriate activation timing during the swing and stance phases of gait (Figure 2), respectively, to reduce ankle muscle coactivation (Figure 4). Monitoring changes in SOL/TA coactivation during FastFES training may therefore serve as an indicator of intervention effectiveness and help with dosing. If and to what extent ankle muscle coactivation is altered by FastFES likely depends on baseline neuromechanical deficits and clinical characteristics of the individual, which warrants further research.

### Limitations

Although we evaluated effects of FastFES across multiple domains (i.e., clinical, biomechanical, muscular), each domain was evaluated during separate sessions across different days. Additionally, gait biomechanics were assessed while walking on a treadmill whereas muscle activity was assessed while walking overground. Therefore, we cannot be certain that observed changes in muscle activity directly caused changes in gait biomechanics. However, the ultimate goal for treadmill-based training interventions such as FastFES is to transfer gains in gait performance to overground walking, and these results show that muscle activity was altered during overground walking following FastFES and accompanied by changes in overground walking performance. The analysis of muscle activity was restricted to the SOL and TA on the paretic limb to focus only on the muscle groups that were stimulated during FastFES. Because changes in recruitment of other muscles likely occurred as well, it will be important to evaluate muscles beyond those that are targeted by the intervention, e.g., using motor module or muscle synergy analysis (31), to provide a more complete understanding of the mechanisms of FastFES-induced improvements neuromuscular control of walking. It is likely that other unmeasured factors such as cognitive engagement during rehabilitation, psychosocial status, stroke lesion volume, corticospinal integrity and excitability, and fatigue could contribute to treatment responses or lack thereof (e.g., in Participant NR1). Consistent with the limitation of a case-series study design, we did not include a control group in this study, each individual served as their own control and timing of ankle muscle activity was qualitatively compared to previously published healthy control data. In similar case-series designs, incorporation of multiple baseline and post-training evaluations would aid with the identification of subject-specific inter-session and physiological variability, providing better indices to evaluate the magnitude of training-induced gait changes. Future studies incorporating a larger cohort of individuals who go through FastFES with appropriate control groups will be necessary to confirm the neuromuscular mechanisms underlying improved clinical walking function through FastFES.

## Conclusions

The findings of this case-series study, albeit preliminary, provide the rationale and foundations for larger-sample studies using similar methodology. By concurrently evaluating muscle activity, biomechanics, and clinical walking function in the same cohort of post-stroke individuals undergoing 18 sessions of FastFES gait training, our preliminary case series study demonstrates that FastFES can improve walking function through improved recruitment of both ankle plantarflexors during stance and the dorsiflexors during swing while walking overground. How the activity of each muscle was altered with FastFES differed between responders (e.g., R1 primarily improved SOL timing whereas R2 improved TA timing), which is consistent with heterogeneity of clinical and biomechanical changes commonly observed following FastFES and other gait interventions. Nevertheless, both responders reduced SOL/TA coactivation after FastFES, suggesting that inducing more appropriate (i.e., reduced) SOL/TA coactivation may be an important neuromuscular mechanism underlying improvements in gait function after FastFES training, and warrants future research in a larger cohort. This suggests the pre-treatment ankle muscle status could be used for inclusion into FastFES gait rehabilitation. Our findings illustrate that analyzing muscle activity can reveal unique insights about neuromuscular mechanisms underlying improved walking function, which cannot be obtained through either clinical or biomechanical measures of gait performance. In addition to providing promising preliminary results regarding the value added by inclusion of muscle activation measures in clinical studies, our data also underscore the complexity of inter-relationships among outcome measures of mobility and function (clinical test scores), gait quality (biomechanics), and neuromuscular coordination (EMG). The multi-factorial processes underlying recovery of post-stroke gait function merit more in-depth investigation.

## Availability of Data and Materials

Data and materials can be made available upon request to the authors.

## Author Contributions

JA, LT, and TK contributed to the conception and design of the study. TK performed the training sessions. TK and JA performed the experiments and collected data. JA, LT, and TK analyzed data and interpreted results. JA prepared figures. All authors were involved in drafting, editing and revising the manuscript, and approved the submitted version.

### Conflict of Interest Statement

The authors declare that the research was conducted in the absence of any commercial or financial relationships that could be construed as a potential conflict of interest.
